# A Comparative Evaluation of Unilateral and Bilateral Sequential Lung Isolation for Vertebral Body Tethering: A Retrospective Propensity Matched Analysis

**DOI:** 10.7759/cureus.59723

**Published:** 2024-05-06

**Authors:** B Randall Brenn, Gregory M Disilvio, Evan Yarnall, Jessica Steindler, Suhail Tarazi, Alexander Rompala, Kyrillos Akhnoukh, Dinesh K Choudhry

**Affiliations:** 1 Anesthesiology, Shriners Children's-Philadelphia, Philadelphia, USA; 2 Anesthesiology/Pediatric Anesthesiology, Vanderbilt University Medical Center, Nashville, USA; 3 Clinical Research, Boston University School of Medicine, Boston, USA; 4 Clinical Research, Temple University Hospital, Philadelphia, USA; 5 Clinical Research, Lake Erie College of Osteopathic Medicine, Erie, USA; 6 Orthopaedics, Monmouth Medical Center, Philadelphia, USA; 7 Clinical Research, Shriners Children's-Philadelphia, Philadelphia, USA; 8 Anesthesiology and Critical Care, Thomas Jefferson University Hospital, Philadelphia, USA

**Keywords:** adolescent, scoliosis, vertebral body tethering, acute lung injury, lung isolation

## Abstract

Background: Vertebral body tethering (VBT) requires a thoracoscopic approach to visualize the vertebral bodies. Lung collapse and re-expansion have the potential to cause acute lung injury, resulting in increased oxygen and ventilation requirements.

Aims: We compared the intraoperative ventilator management, intra- and postoperative blood gas determinations, and hospital stay information between adolescents undergoing unilateral versus bilateral lung isolation for vertebral body tethering.

Methods: A study cohort of 132 propensity-matched cases (66 unilateral and 66 bilateral) was derived from 351 consecutive VBT cases. Patient demographic information, case information, fluid administration, ventilatory settings data, blood gas parameters, and complete blood count and differential data were entered into a datasheet. Derived parameters included values calculated from the alveolar gas equation to develop an oxygen cascade and measures of inflammatory response. Chi-square was used for categorical data, and independent samples and *t-*tests were used for continuous data.

Results: The double lung isolation group required higher peak inspiratory pressures (SL 29±5 vs. DL 31±5, *p*=0.026), resulting in higher tidal volume (SL 246±63 vs. DL 334±101, *p*<0.001) and tidal volume per kg (SL 5.6±1.4 vs. DL 6.9±2, *p*<0.001) as compared to the single lung group. The double lung group required a higher partial pressure of inspired and alveolar oxygen as well as a higher alveolar to arterial oxygen tension gradient (SL 417±126 vs. DL 485±96, *p*=0.001) to achieve optimal arterial oxygen tension. Patients with double lung isolation had similar intensive care lengths of stay but a longer hospital stay than single lung isolation patients.

Conclusion: Patients undergoing double lung isolation required greater ventilatory support and had more evidence of acute lung injury, as evidenced by a higher postoperative alveolar to arterial oxygen gradient; however, these healthy adolescents tolerated the procedure well and only differed in the hospital length of stay by a day.

## Introduction

Vertebral body tethering (VBT) has gained popularity and is now a Food and Drug Administration (FDA) approved alternative to posterior spinal fusion surgery for young adolescents with less than 45-degree spinal curves and further growth potential [1]. Surgical access to the vertebral bodies is achieved using a thoracoscopic approach in the lateral decubitus position to minimize injury to the chest wall and contents of the thoracic cavity [2]. One lung ventilation (OLV) using a double-lumen endotracheal tube or bronchial blocker is necessary for optimal access and visualization via the thoracoscopic approach.

The physiologic changes associated with single-lung ventilation are well described [3]. The blocked, unventilated lung loses its outward expansion forces and collapses to near its residual volume. The dependent, ventilated lung becomes compressed from the weight of the mediastinum and the elevation of the diaphragm by the abdominal contents and, due to gravity, becomes relatively engorged with blood, necessitating positive end-expiratory pressure to expand for adequate gas exchange. The ventilation of the dependent lung and the patient's resultant oxygenation and respiratory status is dependent on the respiratory parameters: tidal volume (VT), respiratory rate (RR), peak inspiratory pressures (PIP), positive end-expiratory pressure (PEEP), and the inspiratory/expiratory time ratio manipulated by the anesthesia team.

The advent of fiber-optic bronchoscopy has led to more effective lung isolation and, with the use of intravenous anesthetic agents, has had little or no detrimental effect on hypoxic pulmonary vasoconstriction. Thus, the risk of hypoxia due to a ventilation/perfusion mismatch is less frequent and has been replaced by the risk of acute lung injury (ALI). The process of collapse and reexpansion of the lung, often with increased inspired oxygen concentrations, may be associated with histological changes consistent with ALI and proinflammatory cytokine release in both the collapsed and ventilated lung [3].

Our institution has been performing bilateral VBT for preadolescent and adolescent patients who have significant thoracic and lumbar spinal curves. This requires a surgical approach via both the right and left chest, one at a time, necessitating each lung to go through the process of collapse and re-expansion, known as sequential bilateral lung isolation (BSLI). In this unique scenario, patients are therefore at increased risk of ALI because both lungs undergo the collapse re-inflation cycle.

The goal of our study was to compare the intraoperative respiratory parameters, laboratory values, and postoperative outcomes of patients receiving unilateral lung collapse and reexpansion for one side VBT versus those receiving bilateral sequential lung collapses and re-expansion for bilateral VBT.

## Materials and methods

Approval was obtained from the Shriners Hospitals for Children Institutional Review Board (IRB) to retrospectively examine the records of all patients undergoing VBT between 2013 and 2019. Due to the retrospective nature of the study, informed consent was waived. All surgeries were performed at the Shriners Hospital for Children-Philadelphia, a specialty orthopedic and spine center in the Mid-Atlantic region of the United States. The data used for examination (demographics, basic surgical case information) was extracted for review from a locally maintained database of all VBTs performed between January 2013 and January 2019. Additional intraoperative and laboratory parameters were obtained from the hospital’s electronic medical record (Powerchart, Cerner Corporation, North Kansas City, MO, USA). Data specific to anesthesia was obtained from the anesthesia module, and laboratory information was obtained from the Results Review sections of the electronic medical record.

**Figure 1:**
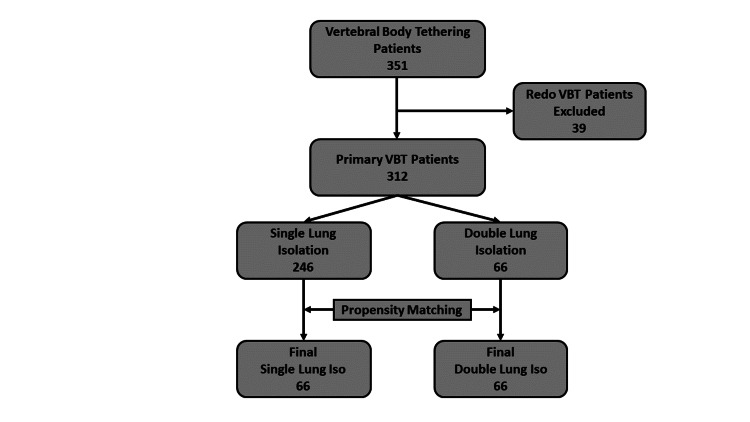
Flow diagram of our cohort selection process.

The initial cohort consisted of all patients undergoing VBT. Patients undergoing redo (repeat) VBT were excluded for the likelihood of significant pleural adhesions complicating the procedure and recovery. The flow diagram of case inclusion is presented in Figure 1.

All patients had American Society of Anesthesiologists physical status II. The basic patient information that we obtained included the age (in months), body weight (in kg), gender, the magnitude of the primary (thoracic) and secondary (lumbar) curves (as measured in degrees), and the number of vertebral levels that were tethered. Intraoperative case information included surgical procedure time and total anesthesia time (in minutes), the estimated blood loss, and crystalloid and albumin (in ml) administered. Derived parameters included the amount of crystalloid and albumin (ml/kg) administered. In addition, information was collected for both the pediatric intensive care unit (PICU LOS) and total hospital length of stay (LOS) in days.

The anesthetic plan consisted of general anesthesia by way of total intravenous anesthesia (TIVA) utilizing propofol and either sufentanil or remifentanil. Muscle relaxation was achieved with rocuronium at induction for placement of a double lumen endotracheal tube (ETT), allowed to wear off, and only given subsequently as needed. Placement of the left bronchial ETT was confirmed by auscultation and fiberoptic bronchoscopy, assuring correct positioning of the bronchial cuff. The patients were placed in the appropriate lateral decubitus positions for the procedure, and carbon dioxide was used for thoracoscopic insufflation.

Respiratory settings data, recorded during the period of single and bilateral lung collapse, were extracted from the anesthesia record. The changes in respiratory settings were made to ensure adequate oxygenation in both groups during the surgical procedure (SpO2 >95, VT = 5-8 ml/kg). These included the highest inspired oxygen fraction administered (%FIO2), the highest peak inspiratory pressure applied (as recorded from the ventilator settings), and the highest average tidal volumes (VT) recorded (ml). While intraoperatively the tidal volumes are measured breath-to-breath, the anesthesia electronic record retrieves an average of these values and presents them at 15-minute intervals. The highest VT per kilogram was derived by dividing the highest average VT by the weight.

Arterial blood gas (ABG) measurements were reliably performed at baseline (before the start of single lung ventilation) and on arrival at the pediatric intensive care unit (PICU). The baseline and lowest arterial oxygen (PaO2), baseline and highest arterial carbon dioxide (PaCO2), and baseline and lowest pH were recorded during the time of lung collapse. By utilizing the alveolar gas equation, it was possible to derive several parameters for comparison, including the inspired oxygen (PIO2) delivered, the alveolar oxygen (PAO2) obtained, and the alveolar-arterial (A-a) gradient on arrival at the PICU. In addition, we recorded the time to room air and whether or not the patient remained on room air in the PICU.

Statistical methods

Descriptive and frequency statistics were used to describe the demographic and clinical characteristics of the sample. Propensity score-based matching (FUZZY method in IBM Corp. Released 2019. IBM SPSS Statistics for Windows, Version 26.0. Armonk, NY: IBM Corp.) was used to match single and double participants based on age, weight, and gender for purposes of analysis. Chi-square analysis was used to compare the matched groups based on categorical parameters. For continuous parameters, the statistical assumptions of normality and homogeneity of variance were checked before choosing a between-subjects test. If both assumptions were met, independent sample t-tests were used to compare the groups on continuous variables. Means and standard deviations were reported and interpreted for the t-test analyses. If either assumption was violated, non-parametric Mann-Whitney U tests were used to compare the groups on ordinal or continuous variables. Statistical significance was assumed at a two-sided alpha value of 0.05. All statistical analyses were performed using IBM Corp. Released 2019. IBM SPSS Statistics for Windows, Version 26.0. Armonk, NY: IBM Corp.

## Results

A total of 351 cases were available for review. Of these, 39 were excluded as they were redoing tethering procedures, and of the remaining 312 cases, 246 patients had single lung isolation (SL) and 66 had double lung isolation (DL). After propensity matching, two groups of 66 patients each were compared (Figure 1).

The comparison of the demographic and case data between the full cohort of SL patients as compared with the DL patients is given in Table 1.

**Table 1:** Demographics of Full and Propensity Matched Cohort Mag 1*: magnitude of the primary thoracic curve; Mag 2*: magnitude of the secondary lumbar curve; SD: standard deviation; †: Mann-Whitney U test

	Full Cohort			Propensity Matched Cohort			
								Single Lung Isolation	Double Lung Isolation		Single Lung Isolation	Double Lung Isolation			
n = 246	n = 66		n = 66	n = 66				
		P-value			P-value			
Age, mean (SD)								
Months	153 (17.1)	154 (14.3)	0.883	154 (17.9)	154 (14.3)	0.949		
Weight, mean (SD)								
Kg	46.8 (11.3)	49.6 (10.5)	0.066	44.8 (9.9)	49.6 (10.5)	0.039		
Gender, n (%)								
Female	207 (84.1)	61 (92.4)	0.09	57 (86.4)	61 (92.4)	0.26		
Male	39 (15.9)	5 (7.6)		9 (13.6)	5 (7.6)			
Mag 1* Curve, mean (SD)								
Degrees (Thoracic)	52.4 (11.3)	53.1 (8.9)	0.642	52.7 (11.1)	53.1 (8.9)	0.794		
Mag 2* Curve, mean (SD)								
Degrees (Lumbar)	33.7 (11.9)	49.9 (8.0)	< .001†	34.2 (13.3)	49.9 (8.0)	< .001†		
Levels Tethered, mean (SD)								
Number	7.86 (0.93)	11.1 (0.71)	< .001†	7.89 (0.99)	11.05 (0.71)	< .001†		

After propensity matching, the groups were very similar in age and gender distribution but differed in weight. The average magnitude of the thoracic curves of the patients was quite similar, but as expected, the average magnitude of the lumbar curves and the number of levels tethered were significantly greater in the DL group (Table 1). 

Patients undergoing sequential double lung isolation had significantly longer surgical and total anesthesia times and lost more blood (in total and per kilogram body weight) than those undergoing single lung isolation. In addition, the amount of crystalloid and albumin administered was greater in the DL group (Table 2). 

**Table 2:** Intraoperative and LOS Parameters Comparison EBL: estimated blood loss; LOS: length of stay; PICU: pediatric intensive care unit

	Single Lung Isolation	Double Lung Isolation	*P-*value
	n=66	n=66	
Surgery Time, mean (SD)			
min	184 (52)	383 (69)	< .001†
Anesthesia Time, mean (SD)			
min	278 (66)	482 (75)	< .001
EBL, mean (SD)			
ml	98 (130)	183 (127)	< .001
EBL/KG, mean (SD)			
ml/kg	2.3 (3.0)	3.9 (3.0)	0.003
Crystalloid, mean (SD)			
ml	1593 (543)	2287 (612)	< .001
Crystalloid/kg, mean (SD)			
ml/kg	36.6 (13.2)	47.6 (14.6)	< .001
Albumin, mean (SD)			
ml	322 (259)	623 (330)	< .001†
Albumin/KG, mean (SD)			
ml/kg	7.5 (6.7)	13.0 (6.9)	< .001

The comparison of intraoperative respiratory support is summarized in Table 3. The highest FIO2 delivered was not significantly different between the two groups. However, the highest average delivered PIP (SL 29±5 vs. DL 31±5, p=0.026), VT (SL 246±63 vs. DL 334±101, p<0.001), and VT/kg (SL 5.6±1.4 vs. DL 6.9±2, p<0.001) were significantly higher in the DL group (Table 3). 

**Table 3:** Ventilatory Parameters during Lung Isolation FIO2: fraction of inspired oxygen; PIP: peak inspiratory pressure; Vt: tidal volume; Vt/Kg: tidal volume per kilogram

	Single Lung Isolation	Double Lung Isolation	*P-*value
	n=66	n=66	
Highest FIO2, mean (SD)	92.9 (14.6)	96.3 (10)	p=0.296
% FIO2			
Highest PIP, mean (SD)	29 (5)	31 (5)	p=0.026
cmH2O			
Highest Vt, mean (SD)	246 (63)	334 (101)	p<0.001
ml			
Highest Vt/Kg, mean (SD)	5.6 (1.40)	6.9 (2)	p<0.001
ml/Kg			

Blood gas data on arrival at the PICU is presented in Table 4. There were no significant differences between the groups with respect to PaO2, PaCO2, or pH. However, there were significant differences noted, as the DL group received higher PIO2 (SL 632±118 vs. DL 687±71, p=0.002) and had a higher calculated PAO2 (SL 574±117 vs. DL 626±72, p=0.003) than the SL group. The calculated A-a gradient was also significantly greater in the DL group (SL 417±126 vs. DL 485±96, p=0.001), while the final PaO2 was similar in both groups. 

**Table 4:** Blood Gas Values on Arrival to PICU, and ICU Outcome Measures PAO2: partial pressure of alveolar oxygen; PaO2: partial pressure of arterial oxygen; PaCO2: partial pressure of arterial carbon dioxide; A-a Difference: alveolar to arterial difference for oxygen; PICU: pediatric intensive care unit; LOS: length of stay

	Single Lung Isolation	Double Lung Isolation	*P-*Value
PAO2, mean (SD)			
mmHg	189 (79)	226 (97)	0.007
PaO2, mean (SD)			
mmHg	119 (33)	136 (69)	0.065
PaCO2, mean (SD)			
mmHg	53 (8)	52 (6)	0.272
A-a Difference, mean (SD)			
mmHg	65 (57)	86 (77)	0.019
Time to Room Air, mean (SD)			
min	1321 (459)	1443 (741)	0.254
Stayed on Room Air, % (n)			
Yes	56 (37)	36 (24)	0.023
No	44 (29)	64 (42)	
PICU LOS, mean (SD)			
days	1.41 (0.63)	1.43 (0.63)	0.837
Hospital LOS, mean (SD)			
days	4.5 (1.0)	5.2 (1.9)	.026†

PICU stay information is also shown in Table 4. In the PICU, while the time to commence room air did not differ between the groups, the ability of patients to remain on RA differed, with more DL patients failing the initial room air challenge (SpO2<95%) than SL patients (DL 42% vs. SL 29%, p=0.023). Additionally, while there were no differences seen in the PICU length of stay, DL patients did require a longer stay in the hospital (4.5±0.96 vs. 5.2±1.87 days, p=0.026). 

## Discussion

To our knowledge, this is the first study comparing the anesthetic management of adolescents undergoing single lung isolation versus sequential double lung isolation as part of an elective procedure. SL isolation is used quite commonly, but DL isolation involving sequential collapse and reexpansion of both lungs is relatively uncommon and, therefore, unexamined to date. While the technique and hazards associated with collapsing a single lung are well known [3], the respiratory effects associated with sequentially collapsing both lungs have not been evaluated.

This retrospective comparison of the perioperative course of adolescents undergoing either SL or DL isolation demonstrates that although intraoperative blood gas profiles are similar, there are significant differences in ventilator management to achieve this, with notable differences in oxygen delivery. Specifically, we found that the lack of differences in blood gas values such as PaO2, PaCO2, or pH was generally attributed to increased ventilatory pressures received by DL patients and their resultant increases in tidal volumes to achieve close to normal blood gas values. 

On arrival in the PICU, while the arterial oxygen tension was ultimately similar, the administered inspired oxygen and the calculated alveolar oxygen tension were higher, with a significantly increased A-a gradient in the DL group, indicating a higher shunt fraction.

The literature regarding patient outcomes after VBT has largely focused on the success of spinal correction and less on thoracoscopy and lung collapse. The technique of using thoracoscopy for VBT is well described [2]; however, there are recent reports regarding respiratory complications. One study of 56 patients documented five complications: two patients with persistent lung atelectasis, one with lobar atelectasis, one with chylothorax, and one with pleural effusion [4]. A smaller series of 21 patients revealed that one developed a chylothorax [5]. In a series of 57 VBT patients, perioperative pulmonary complications included three patients with persistent atelectasis, one patient with a superficial thoracic wound infection, and one patient who developed pneumonia [6]. Complications from OLV such as these are not unexpected and have been easily treatable. The literature on long-term pulmonary complications is, as of yet, unavailable.

On the other hand, literature about the use of BSLI is rare and exists only as case reports. There are case reports of sequential lung isolation with a unique device called the EZ blocker (Teleflex, Reading, PA). It has been used in the case of a 28-year-old male undergoing BSLI for thoracoscopic treatment of hyperhidrosis via bilateral dorsal sympathectomy [7]. In addition, there is a case report of a 51-year-old woman with an anterior mediastinal mass, where this device was used because of a need to collapse the right lung and then subsequently the left. The use of this device and BSLI, however, was unplanned [8]. The value of the EZ blocker device is that it can be used in situations where the need for OLV (or BSLI) is unexpected. There is no literature to our knowledge on the planned use of BSLI with double-lumen tubes on a planned elective basis.

In his review, Lohser outlined the various mechanisms of lung injury associated with OLV. Specifically, the dependent, ventilated lung can suffer volutrauma from over-distention, atelectrauma from low-volume ventilation, capillary shear stress from hyperperfusion, biotrauma from inflammation, and oxidative injury from high oxygen concentrations. The collapsed lung may suffer from atelectasis followed by recruitment at the end of the procedure, hypoperfusion and ischemia followed by reperfusion injury, biotrauma, and surgical trauma [3]. What is unique about our DL population is that both lungs sequentially undergo collapse followed by an expansion cycle. Hence, it would not be unreasonable to surmise that the DL group could potentially have more pulmonary complications or greater inflammation than the SL group.

In adults, protective ventilation in esophagectomy patients has been shown to reduce inflammatory mechanisms, improve lung function, and lead to earlier extubation [9]. Sevoflurane has been shown to reduce inflammatory mediators and lead to significantly better clinical outcomes [10]. In our VBT population, we are not able to use sevoflurane for this benefit, as total intravenous anesthesia (TIVA) with propofol and narcotics is standard for neurophysiologic monitoring. In laboratory animals, a preceding OLV alveolar recruitment regimen improved dependent lung aeration, increased PAO2, and reduced mechanical stress [11].

There is evidence that methylprednisolone reduces inflammatory markers and increases anti-inflammatory markers in adolescents undergoing OLV. Interleukin-6 was lower at six and eighteen hours after steroid administration, while Interleukin-10 was higher six hours after treatment [12]. The Nuclear Factor-kB pathway has also been implicated in the development of lung injury during OLV, and in an animal model, this pathway can be inhibited, producing a protective effect [13].

The major limitations of this study are its retrospective design and reliance on the extraction of information from electronic medical records. Fortunately, the ventilatory parameters were digitally collected and could be retrieved for analysis. However, there was no ability to control the ventilatory regimen, the recruitment maneuvers, or the precise timing of laboratory samples.

## Conclusions

Bilateral VBT is the only elective procedure that requires BSLI. As compared with SL isolation, there is greater shunting in the DL group since it requires establishing ventilation in a previously collapsed lung. The ventilatory parameters used to maintain similar oxygenation and ventilation are thus greater, and there is likely more inflammation due to the collapse and reexpansion of both lungs. While we have documented oxygenation and ventilation differences between SL and DL patients overall, healthy adolescents have done well and, to date, have had uneventful postoperative recoveries.
